# Baseline and 7-Year longitudinal trajectories of systolic blood pressure and all-cause mortality in the elderly: A large prospective cohort study in China, 2017–2023

**DOI:** 10.3934/publichealth.2025043

**Published:** 2025-08-29

**Authors:** Zhuo Wang, Yujin He, Tianyong Wu, Zifang Zhou, Zhi Lei, Rujun Liao, Fangyi Yu, Pengyuan Yang, Xiaoyan Wang, Ling Liu, Longling Yang, Qing Yang, Xuefeng Tang, Xianping Wu, Bo Zhong, Hang Chen, Peng Yin

**Affiliations:** 1 Department of Chronic and Non-communicable Disease Control and Prevention, Sichuan Center for Disease Control and Prevention, Chengdu, China; 2 Department of Chronic and Non-communicable Disease Control and Prevention, Luzhou Center for Disease Control and Prevention, Luzhou, China; 3 National Center for Chronic and Non-communicable Disease Control and Prevention, Chinese Center for Disease Control and Prevention, Beijing, China; 4 School of Public Health, North Sichuan Medical College, Nanchong, China

**Keywords:** systolic blood pressure, elderly, mortality, Chinese, prospective cohort

## Abstract

**Background:**

Hypertension is a critical modifiable risk factor for mortality in the elderly, yet optimal systolic blood pressure (SBP) targets for older adults remain unclear. This study examined the association between SBP trajectories and all-cause mortality in Chinese elderly individuals, aiming to identify age-adjusted SBP ranges for survival benefits.

**Methods:**

A population-based prospective cohort study was conducted in Luzhou, China (2017–2023). A total of 390,100 participants aged ≥65 years were followed for 1,994,050 person-years, with 48,013 deaths analyzed. Cox proportional hazards and restricted cubic spline (RCS) Cox regression models were used to evaluate baseline SBP categories, 7-year mean SBP, and longitudinal trajectories (Class 1: ideal to elevated; Class 2: normal-high to elevated; Class 3: mild hypertension to elevated).

**Results:**

Baseline SBP: Lowest mortality risk at 100–139 mmHg (HR < 1). 7-year mean SBP: Optimal range at 120–159 mmHg. Trajectories: Class 2 (normal-high to elevated) had the lowest all-cause mortality (HR: 0.90, 95% CI: 0.88–0.92). Class 1 showed the lowest cardiovascular mortality. Class 3 exhibited the lowest non-cardiovascular/cancer mortality.

**Conclusions:**

Gradual SBP increases from normal (≤160 mmHg) with age are associated with reduced mortality in the elderly. These findings challenge strict hypertension control guidelines, advocating for age-adjusted SBP targets to optimize survival outcomes. Further validation across different ethnic groups and regions will be needed in the future.

## 1. Introduction

With increasing age, arteriosclerosis causes an increase in systolic blood pressure (SBP), which is an important predictor of cardiovascular disease (CVD) events and all-cause mortality, and a proper indicator of CVD risk in the elderly [1]. Therefore, achieving optimal blood pressure control is an important healthcare measure and challenge. However, the target blood pressure value, especially for the elderly, is still controversial. With the intensification of global aging, the burden of diseases related to hypertension levels is increasing, and more evidence is needed to support the appropriate levels of SBP in the elderly population [2].

Some clinical reports indicate that in order to reduce the incidence of cardiovascular events, older patients with hypertension need to control systolic blood pressure to 110–130 mmHg or less [3],[4]. However, there are also reports that strengthened blood pressure control leads to a higher incidence of adverse events, such as hypotension, syncope, electrolyte abnormalities, and acute kidney injury or failure, in older adults [5],[6]. In addition, it has been reported that the long-term trajectory of blood pressure in the elderly is also closely related to their underlying health. As long as systolic blood pressure is not chronically higher than 180 mmHg, it will not increase cognitive ability, dementia risk, and all-cause mortality in the elderly [7]. Due to the coexistence of multiple diseases in the elderly, strict control of SBP in the elderly may not reduce the risk of fatal and non-fatal major cardiovascular events [8]. In addition, appropriate blood pressure is necessary to ensure the nutritional needs of all organs in the body. Each organ may have different needs for blood pressure levels, so appropriate blood pressure levels should not be established simply based on individual organ protection (such as cerebrovascular protection) [9]. Currently, there is still controversy regarding the level of SBP and its development trajectory in the elderly without significantly increasing the risk of serious adverse events [10]–[12]. Therefore, more evidence is needed to comprehensively reflect blood pressure and health outcomes in the elderly.

Therefore, we aim to conduct a cohort study using a 7-year natural population physical examination data from Luzhou, China, to test the correlation between baseline SBP and longitudinal SBP trajectories and overall mortality risk.

## 2. Materials and methods

### 2.1. Data source and study population

The Luzhou Elderly Cohort is a longitudinal cohort study conducted in Luzhou city, in western China, with a population of approximately 5 million, covering all elderly populations. From 2017 to 2023, elderly individuals could receive one free physical examination per year for seven consecutive years, i.e., one follow-up visit. The collected data items are in accordance with the requirements of basic public health services carried out in China [13]. Face-to-face interviews were carried out according to a standardized protocol to collect demographics and lifestyle information for all participants aged 65 years and older. Physical examinations were administered, and blood samples were taken after 24 hours of fasting by trained professionals.

**Figure 1.**
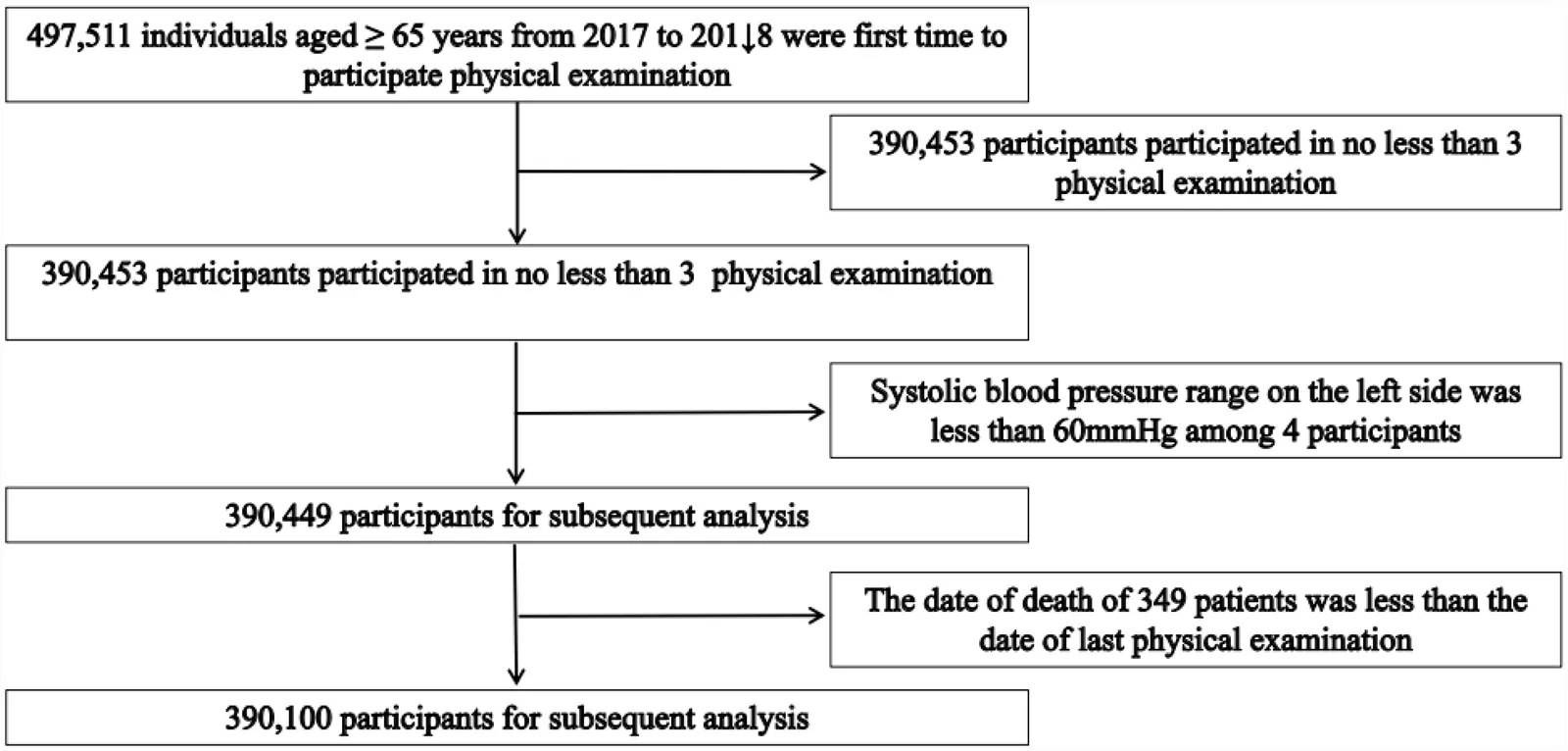
The study participants' inclusion and exclusion process.

In this study, 497,511 participants from 2017 to 2018 were initially included. We excluded participants who had less than 3 examinations (N = 390,453), whose systolic blood pressure on the left side was less than 60 mmHg (N = 4), and whose date of death was earlier than the date of the last physical examination (N = 349). Finally, 390,100 elderly people aged 65 years or older participated in the final analysis (Figure 1).

This study was approved by the Luzhou Center for Disease Control and Prevention Ethics Review Committee (LZCDC/LS-2023001), and all participants provided informed consent.

### 2.2. SBP measurement and covariates

Blood pressure was measured twice in the left arm using calibrated upper-arm electronic sphygmomanometers by a trained doctor according to standard protocols. Participants sat in a quiet and comfortable temperature room after resting for at least 5 minutes. If the difference between the two measurements was greater than 10 mmHg, a third measurement was conducted, and the average value was used in further analysis.

Information on age, gender, marital status (unmarried, married, widowed, divorced), educational level (illiterate, primary or middle or high school, college or above, unknown), tobacco smoking (current, former, never), alcohol drinking (everyday, often, occasionally, never), and physical activity (less than once per week, more than once per week, everyday) was obtained through questionnaires. Height, weight, waist circumference, total cholesterol, and fasting blood glucose were also measured or tested by professional doctors (Table S1).

### 2.3. Mortality and follow-up

The vital status of participants was obtained by linking with the China Population Death Information Registration System. This is a well-established system that collects all deaths occurring in mainland China, including hospital deaths and otherwise. Strict quality control measures were in place to ensure the completeness and accuracy of the reported death cases [14]. The unique national IDs of all the participants were linked with the China Population Death Information Registration System, and the date and cause of death of the deceased were obtained. The end date of follow-up was the date of death or December 31, 2023, whichever came first. Participants who died from any cause were included in the outcome analysis.

### 2.4. Statistical analysis

The study population was divided into six groups according to the observed baseline SBP (<100, 100–119, 120–139, 140–159, 160–179, and ≥180 mmHg). According to the baseline SBP classification and the 7-year average SBP classification, the longitudinal SBP trajectory was divided into three categories: blood pressure gradually increases from ideal values with age; blood pressure gradually increases from normal-high values with age; and blood pressure gradually increases from mild hypertension with age (Figure S1). According to the data type, participants were classified according to baseline SBP and 7-year SBP trajectories, and their characteristics are represented as the mean ± SD of continuous variables and n (%) of categorical variables. The mortality rate was calculated by dividing the number of deaths by the total number of follow-up durations.

A Kaplan-Meier curve and logarithmic rank test were used to compare the differences in mortality rates among elderly individuals grouped according to the 7-year SBP trajectory. Cox proportional hazards regression model was used to estimate the risk ratios (HRs) and 95% confidence intervals (CIs) of all-cause mortality in the baseline SBP groups and 7-year SBP trajectory groups, as well as different baseline and trajectory combinations, using survival days as the time scale, gradually adjusting for potential confounding factors in the multivariate Cox model: sex, age, marital status, educational level, tobacco smoking, alcohol drinking, physical activity, 7-year average waist circumference, 7-year average fasting blood glucose, 7-year average total cholesterol, and 7-year average body mass index [15]. We used a restricted cubic spline (RCS) Cox regression model to examine the association between baseline SBP, 7-year mean SBP, and mortality on a continuous scale, with or without age groups [16]. The 7-year average values of continuous variables are the mean of multiple measurements obtained from elderly individuals during the 7-year follow-up period.

All statistical analyses were performed using R software version 4.3.2 (2023–10–23). All statistical tests were two-sided, and P < 0.05 was considered statistically significant.

## 3. Results

### 3.1. Baseline characteristics and follow-up

This study was based on the elderly cohort in Luzhou, China (2017–2023). A total of 390,100 elderly individuals aged ≥65 years were included. Baseline systolic blood pressure (SBP), 7-year average SBP, and data on longitudinal SBP trajectories (3 trajectory classes: gradual increase from ideal values to elevated levels, gradual increase from normal-high values to elevated levels, and gradual increase from mild hypertension to elevated levels) were collected through annual physical examinations. Outcomes were tracked using the Chinese Population Death Information Registration System, with a total follow-up of 1,994,050 person-years. The associations between SBP and all-cause and categorized mortality rates were analyzed.

Participants' characteristics stratified by baseline SBP and 7-year SBP trajectories are presented in Table 1. The overall study population had a mean ± SD age of 71.34 ± 5.72 years at baseline, with 46.7% being men. Most participants were married (63.04%), had a primary/middle/high school education (52.71%), never smoked (72.18%), never drank alcohol (79.34%), and reported no regular exercise (84.1%). Baseline measurements showed mean ± SD values for waist circumference (WC) of 80.30 ± 9.17 cm, fasting blood glucose (FBG) of 5.77 ± 1.90 mmol/L, total cholesterol (TC) of 5.29 ± 23.03 mmol/L, and body mass index (BMI) of 23.29 ± 44.61 kg/m².

Baseline SBP was categorized into six groups, with the largest proportion in the 120–139 mmHg range (47.57%), followed by 140–159 mmHg (21.79%). Regarding 7-year SBP trajectories, 48.75% of participants showed a gradual increase from normal-high values with age (Class 2), 35.82% from ideal values (Class 1), and 15.4% from mild hypertension (Class 3) (Figures S1 and S2).

During 1,994,050.42 person-years of follow-up, 48,013 deaths were recorded, including 19,302 from cardiovascular disease, 10,095 from cancer, and 18,616 from other non-cardiovascular/non-cancer diseases. The corresponding mortality rates are detailed in Table 2 and Supplementary Tables S2–S4.

### 3.2. Relationship between continuous baseline SBP, 7-year mean SBP, and all-cause mortality

**Figure 2.**
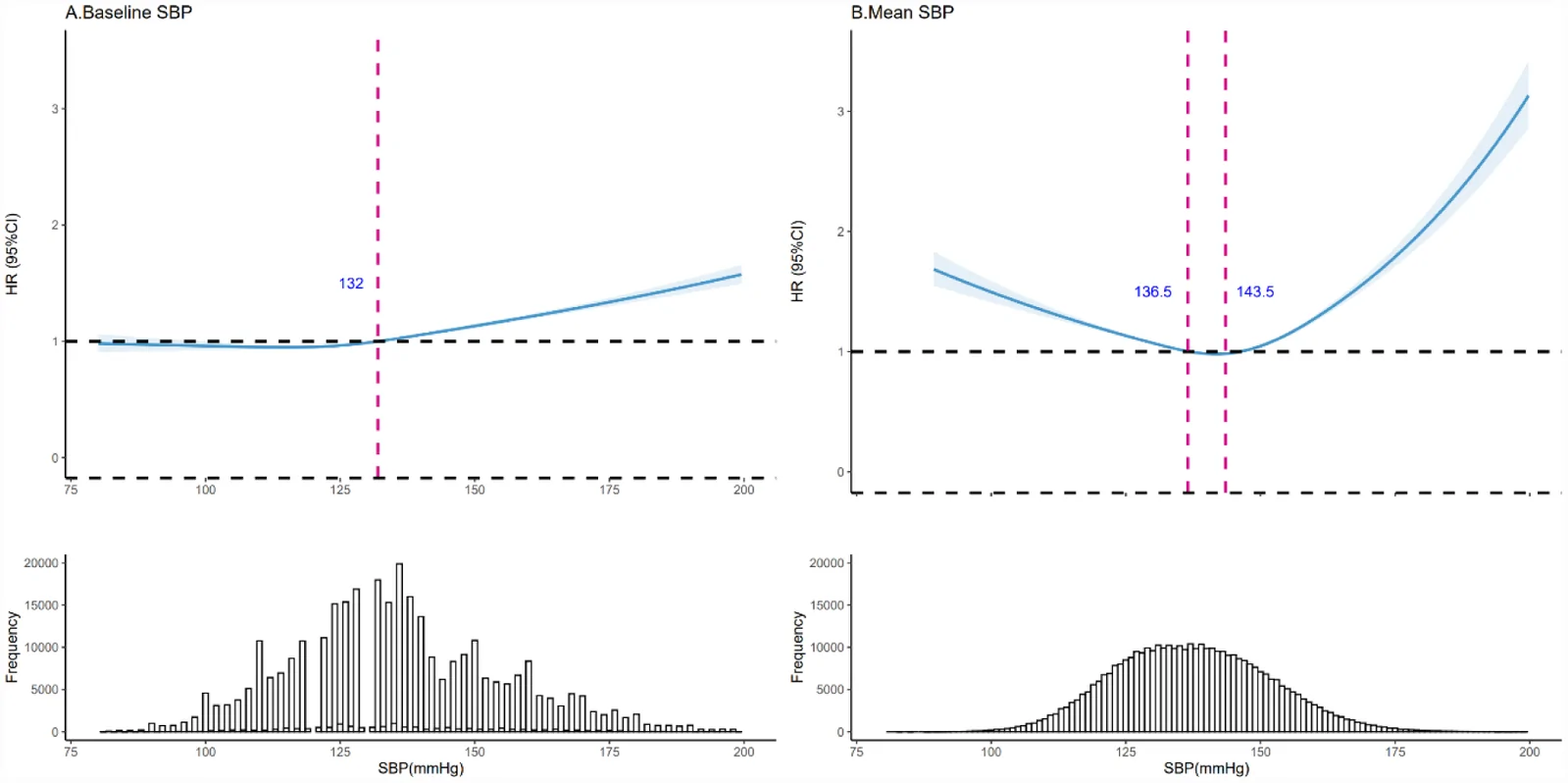
Association of baseline SBP and 7-year mean SBP as a continuous scale with mortality. Note: HRs (solid line) and 95% CIs (shadow) from Cox proportional hazards regression models using restricted cubic splines. Graphs were truncated at 80 and 200 mmHg, as more than 96% of participants were located between these cut points. Abbreviations: SBP = systolic blood pressure; CI = confidence interval.

Restricted cubic spline (RCS) regression revealed nonlinear associations (P < 0.001) between baseline SBP, 7-year mean SBP, and all-cause mortality. For baseline SBP, mortality risk was lowest when SBP was below 132 mmHg (HR < 1) (Figure 2A). For 7-year mean SBP, the lowest risk occurred at 136–144 mmHg (HR < 1) (Figure 2B). After adjusting for covariates (marital status, education, lifestyle factors, and 7-year averages of WC, TC, FBG, and BMI), the optimal ranges shifted slightly to 107–132 mmHg for baseline SBP and 137–146 mmHg for 7-year mean SBP (Figure S3).

**Figure 3.**
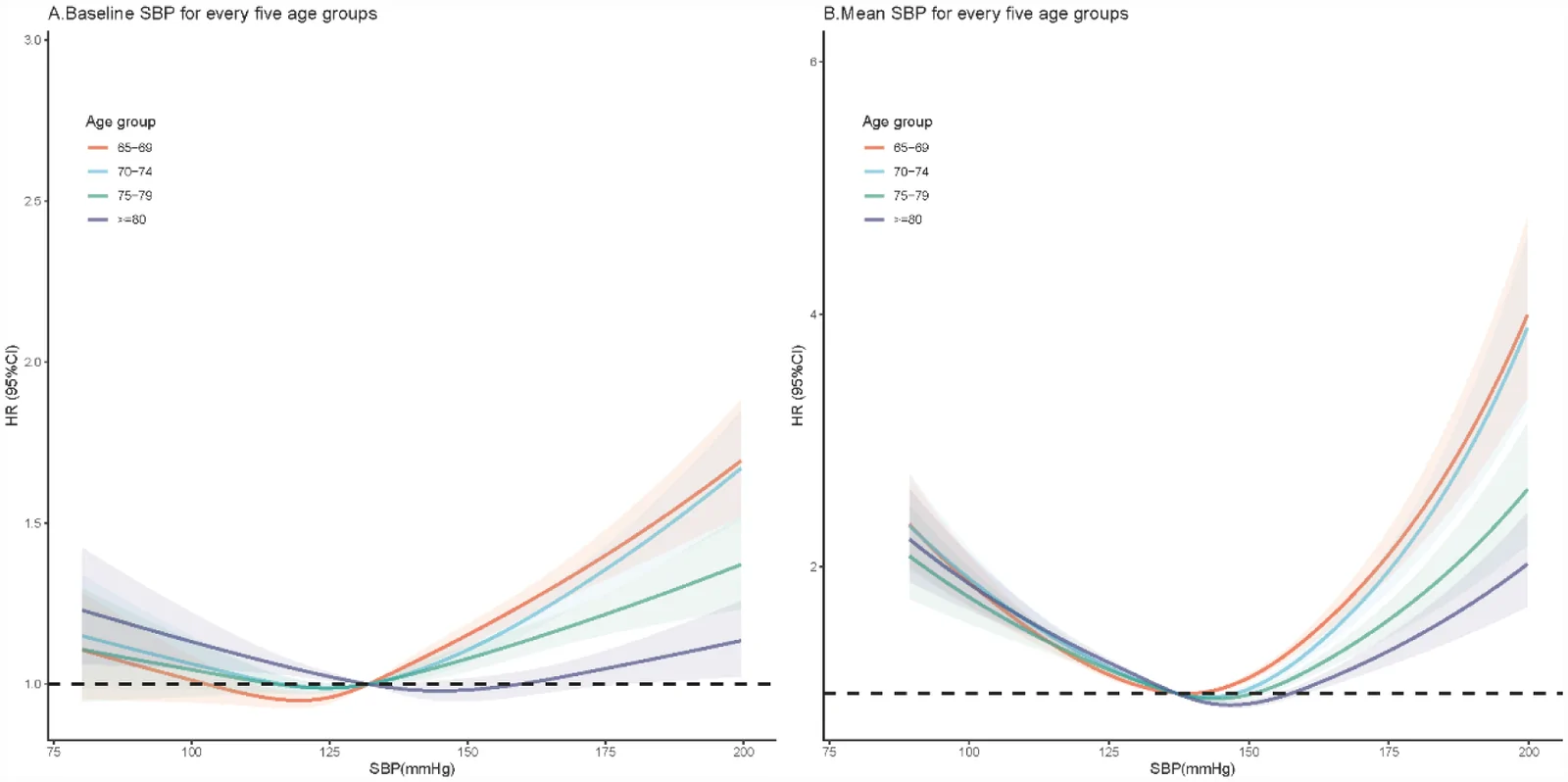
Association of baseline and 7-year mean SBP as continuous variables with mortality in the elderly aged 65–79, 70–74, 75–79, and over 80 years old. Note: HRs (solid line) and 95% CIs (shadow) from Cox proportional hazards regression models using restricted cubic splines. Graphs were truncated at 80 and 200 mmHg, as more than 96% of participants were located between these cut points. Abbreviations: SBP = systolic blood pressure; CI = confidence interval.

Age-stratified analyses (65–79, 70–74, 75–79, and ≥80 years) showed age-specific optimal SBP ranges. For baseline SBP, the lowest mortality risk occurred at 102–132 mmHg (65–79 years), 116–132 mmHg (70–74 years), 115–132 mmHg (75–79 years), and 132–159 mmHg (≥80 years) (Figure 3A). Corresponding ranges for 7-year mean SBP were 137–141 mmHg, 137–148 mmHg, 137–151 mmHg, and 137–158 mmHg, respectively (Figure 3B).

**Table 1.** Baseline characteristics of participants according to SBP.

	Baseline SBP (mmHg)						7-year SBP trajectories			Total
	Grade 1	Grade 2	Grade 3	Grade 4	Grade 5	Grade 6	Class 1	Class 2	Class 3	
**Number of participants**	6448	65,804	185,552	85,022	38,731	8543	139,821	190,193	60,086	390,100
**Age (mean ± SD, years)**	70.62 ± 5.70	70.63 ± 5.55	71.26 ± 5.69	71.71 ± 5.74	72.04 ± 5.85	72.31 ± 5.95	71.80 ± 6.11	71.27 ± 5.57	70.51 ± 5.10	71.34 ± 5.72
**Men (n,%)**	2756 (42.74)	29,806 (45.30)	87,052 (46.92)	40,236 (47.32)	18,290 (47.22)	4043 (47.33)	67,265 (48.11)	88,344 (46.45)	26,574 (44.23)	182,183 (46.70)
**Marriage (n,%)**										
Unmarried	157 (2.43)	1537 (2.34)	4818 (2.60)	2121 (2.49)	1099 (2.84)	292 (3.42)	3420 (2.45)	4877 (2.56)	1727 (2.87)	10,024 (2.57)
Married	4203 (65.18)	43,486 (66.09)	117,511 (63.33)	52,590 (61.86)	23,202 (59.91)	4925 (57.65)	89,526 (64.03)	118,855 (62.49)	37,536 (62.47)	245,917 (63.04)
Widowed	2049 (31.78)	20,402 (31.01)	62,165 (33.50)	29,882 (35.15)	14,240 (36.77)	3287 (38.48)	46,080 (32.96)	65,428 (34.40)	20,517 (34.15)	132,025 (33.85)
Divorced	33 (0.51)	307 (0.47)	845 (0.46)	338 (0.40)	151 (0.39)	31 (0.36)	623 (0.45)	832 (0.44)	250 (0.42)	1705 (0.44)
**Education (n,%)**										
Unknown	54 (0.84)	579 (0.88)	1567 (0.84)	692 (0.81)	320 (0.83)	70 (0.82)	1241 (0.89)	1593 (0.84)	448 (0.75)	3282 (0.84)
College or above	40 (0.62)	405 (0.62)	1126 (0.61)	461 (0.54)	143 (0.37)	23 (0.27)	989 (0.71)	1001 (0.53)	208 (0.35)	2198 (0.56)
Primary, middle, or High school	3186 (49.41)	34,412 (52.29)	99,024 (53.37)	45,198 (53.16)	19,658 (50.76)	4152 (48.60)	73,235 (52.38)	102,052 (53.66)	30,343 (50.50)	205,630 (52.71)
Illiterate	3168 (49.13)	30,408 (46.21)	83,835 (45.18)	38,671 (45.48)	18,610 (48.05)	4298 (50.31)	64,356 (46.03)	85,547 (44.98)	29,087 (48.41)	178,990 (45.88)
**Tobacco smoking (n,%)**
Never	4604 (71.40)	47,288 (71.86)	134,241 (72.35)	61,390 (72.20)	27,912 (72.07)	6130 (71.75)	98,786 (70.65)	138,337 (72.74)	44,442 (73.96)	281,565 (72.18)
Former	253 (3.92)	2138 (3.25)	6167 (3.32)	3159 (3.72)	1317 (3.40)	266 (3.11)	4873 (3.49)	6586 (3.46)	1841 (3.06)	13,300 (3.41)
Current	1585 (24.58)	16,321 (24.80)	44,975 (24.24)	20,410 (24.01)	9462 (24.43)	2141 (25.06)	36,037 (25.77)	45,101 (23.71)	13,756 (22.89)	94,894 (24.33)
**Alcohol drinking (n,%)**										
Never	5328 (82.63)	53,193 (80.84)	147,138 (79.30)	66,963 (78.76)	30,301 (78.24)	6574 (76.95)	111,732 (79.91)	150,424 (79.09)	47,341 (78.79)	309,497 (79.34)
Occasionally	650 (10.08)	6681 (10.15)	19,144 (10.32)	8984 (10.57)	3963 (10.23)	927 (10.85)	14,733 (10.54)	19,635 (10.32)	5981 (9.95)	40,349 (10.34)
Often	128 (1.99)	1558 (2.37)	5052 (2.72)	2279 (2.68)	1094 (2.82)	246 (2.88)	3439 (2.46)	5114 (2.69)	1804 (3.00)	10,357 (2.65)
Everyday	305 (4.73)	4017 (6.10)	13,095 (7.06)	6279 (7.39)	3093 (7.99)	741 (8.67)	9106 (6.51)	13,811 (7.26)	4613 (7.68)	27,530 (7.06)
**Physical activity (n,%)**										
Everyday	640 (9.93)	6900 (10.49)	19,139 (10.31)	10,443 (12.28)	4222 (10.90)	680 (7.96)	14,445 (10.33)	21,436 (11.27)	6143 (10.22)	42,024 (10.77)
More than once/week	231 (3.58)	2192 (3.33)	6265 (3.38)	3199 (3.76)	1311 (3.38)	279 (3.27)	4465 (3.19)	6709 (3.53)	2303 (3.83)	13,477 (3.45)
Less than once/week	90 (1.40)	1021 (1.55)	2918 (1.57)	1404 (1.65)	535 (1.38)	153 (1.79)	2099 (1.50)	3080 (1.62)	942 (1.57)	6121 (1.57)
No exercise	5480 (84.99)	55,622 (84.53)	157,047 (84.64)	69,898 (82.21)	32,616 (84.21)	7423 (86.89)	118,675 (84.88)	158,767 (83.48)	50,644 (84.29)	328,086 (84.10)
**WC (mean ± SD, cm)**	75.78 (8.71)	78.16 (8.75)	80.05 (8.95)	81.86 (9.35)	82.10 (9.41)	82.07 (9.43)	78.31 (8.79)	81.16 (9.17)	82.24 (9.22)	80.30 (9.17)
**FBG (mean ± SD, mmol/l)**	5.53 (1.71)	5.65 (1.79)	5.74 (1.85)	5.90 (2.01)	5.87 (2.02)	5.88 (2.04)	5.63 (1.72)	5.83 (1.95)	5.91 (2.08)	5.77 (1.90)
**TC (mean ± SD, mmol/l)**	4.85 (2.76)	5.13 (8.19)	5.31 (28.97)	5.30 (17.51)	5.47 (23.25)	5.34 (5.61)	5.17 (20.50)	5.35 (25.87)	5.35 (18.63)	5.29 (23.03)
**BMI (mean ± SD, kg/m²)**	21.31 (3.68)	22.36 (15.49)	23.13 (18.98)	23.88 (20.06)	23.91 (13.06)	26.94 (276.40)	22.34 (15.52)	23.62 (20.07)	24.48 (105.27)	23.29 (44.61)

Note: Abbreviations: SBP = systolic blood pressure; SD = standard deviation; WC = waist circumference; FBG = fasting blood glucose; TC = total cholesterol; BMI = body mass index. Grade 1 ≤ 100 mmHg; Grade 2= 100–119 mmHg; Grade 3 = 120–139 mmHg; Grade 4 = 140–159 mmHg; Grade 5 = 160–179 mmHg; Grade 6 ≥ 180 mmHg. Class 1 = Blood pressure gradually increases from ideal values with age; Class 2 = Blood pressure gradually increases from normal-high values with age; Class 3 = Blood pressure gradually increases from mild hypertension with age.

**Table 2.** Association of baseline SBP and 7-year SBP trajectories with mortality.

Group	Number of deaths	Follow-up duration (person-years)	Mortality rate (per 10,000 person-years)	Adjusted HR (95% CI)			
				Model 1^a^	Model 2^b^	Model 3^c^	Model 4^d^
Baseline SBP (mmHg)							
Grade1	762	32,431.31	23.50	NA	NA	NA	NA
Grade2	6887	333,512.47	20.65	0.87 (0.81–0.94)**	0.86 (0.8–0.93)**	0.9 (0.83–0.97)*	0.91 (0.84–0.98)*
Grade3	21,710	947,961.46	22.90	0.93 (0.87–1)	0.86 (0.8–0.92)**	0.91 (0.85–0.98)*	0.95 (0.89–1.03)
Grade4	11,277	437,547.32	25.77	1.03 (0.96–1.11)	0.9 (0.84–0.97)*	0.99 (0.92–1.07)	1.07 (0.99–1.15)
Grade5	5838	198,981.74	29.34	1.16 (1.08–1.26)**	0.99 (0.92–1.07)	1.08 (1–1.17)*	1.17 (1.08–1.26)**
Grade6	1539	43,616.12	35.29	1.41 (1.29–1.53)**	1.18 (1.08–1.28)**	1.28 (1.18–1.4) **	1.38 (1.26–1.51)**
7-year SBP trajectories							
Class 1	18,438	701,689.26	26.28	NA	NA	NA	NA
Class 2	22,416	980,183.86	22.87	0.85 (0.83–0.87)**	0.92 (0.9–0.94)**	0.96 (0.94–0.98)**	0.9 (0.88–0.92)**
Class 3	7159	312,177.3	22.93	0.86 (0.83–0.88)**	1.03 (1–1.06)*	1.1 (1.06–1.13)**	0.93 (0.9–0.96)**

Note: Abbreviations: SBP = blood pressure; HR = hazard ratio; CI = confidence interval. Grade 1 ≤ 100 mmHg; Grade 2 = 100–119 mmHg; Grade 3 = 120–139 mmHg; Grade 4 = 140–159 mmHg; Grade 5 = 160–179 mmHg; Grade 6 ≥ 180 mmHg. Class 1 = Blood pressure gradually increases from ideal values with age; Class 2 = Blood pressure gradually increases from normal-high values with age; Class 3 = Blood pressure gradually increases from mild hypertension with age. ^a^Model 1 was a univariate analysis. ^b^Model 2 was adjusted for sex and age. ^c^Model 3 included variables in Model 1 and was further adjusted for marital status, educational level, tobacco smoking, alcohol drinking, physical activity, 7-year average waist circumference, 7-year average fasting blood glucose, 7-year average total cholesterol, and 7-year average body mass index. ^d^Model 4 included variables in Model 2 and was further adjusted for SBP trajectories or SBP at baseline. *P < 0.05; **P < 0.01.

### 3.3. Associations between baseline SBP, 7-year SBP trajectories, and all-cause mortality

Table 2 shows that compared with the reference group (baseline SBP < 100 mmHg), mortality risk was increased for SBP < 100 mmHg or >160 mmHg in both unadjusted and adjusted models. Among 7-year SBP trajectories, Class 2 (normal-high to elevated) had the lowest all-cause mortality risk (HR: 0.90, 95% CI: 0.88–0.92) after full adjustment for covariates and baseline SBP. Additionally, baseline SBP of 100–119 mmHg (Grade 2) was associated with lower mortality (HR: 0.91, 95% CI: 0.84–0.98) in the fully adjusted model.

Combined analyses of baseline SBP and trajectories (Table 3) showed reduced mortality risk in several combinations, including Grade 2 baseline SBP with Class 2 trajectory, and Grade 3 baseline SBP with Class 2 or 3 trajectories, compared with the reference (Grade 1 + Class 1).

### 3.4. Combined all-cause mortality risk of all variables

**Figure 4.**
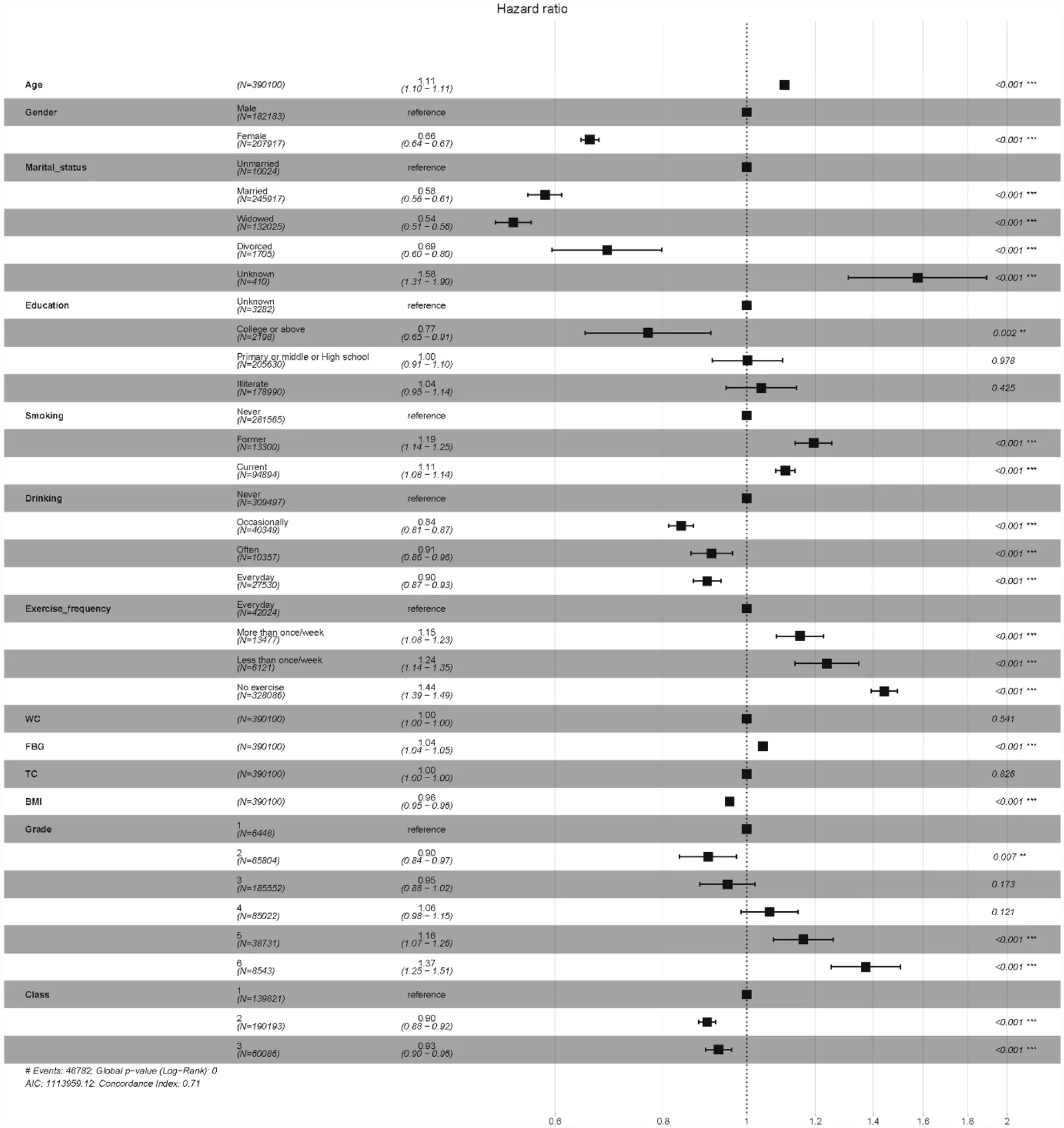
Hazard ratio for the combined associations of age, gender, marital status, educational level, tobacco smoking, alcohol drinking, physical activity, 7-year average WC, 7-year average FBG, 7-year average TC, 7-year average BMI, baseline SBP grade, and 7-year SBP trajectories with all-cause mortality. Note: Class 1 = Blood pressure gradually increases from ideal values with age; Class 2 = Blood pressure gradually increases from normal-high values with age; Class 3 = Blood pressure gradually increases from mild hypertension with age. Grade 1 ≤ 100 mmHg; Grade 2 = 100–119 mmHg; Grade 3 = 120–139 mmHg; Grade 4 = 140–159 mmHg; Grade 5 = 160–179 mmHg; Grade 6 ≥ 180 mmHg. Abbreviations: SBP = systolic blood pressure; WC = waist circumference; FBG = fasting blood glucose; TC = total cholesterol; BMI = body mass index; HR = hazard ratio.

The multivariable risk model (Figure 4) identified factors associated with higher mortality: older age, male sex, unmarried status, smoking, never drinking alcohol, elevated FBG, and baseline SBP > 160 mmHg. Lower mortality was associated with female sex, married status, college education or above, never smoking, occasional drinking, daily exercise, lower BMI, baseline SBP Grade 2, and 7-year SBP trajectories Class 2 and 3.

**Table 3.** Association of different combinations of baseline SBP and 7-year SBP trajectories with mortality.

Group (Grade + class)	Number of deaths	Follow-up duration (person-years)	Mortality rate (per 10,000 person-years)	Adjusted HR (95% CI)		
				Model 1^a^	Model 2^b^	Model 3^c^
1 + 1	702	29,399.91	23.88	NA	NA	NA
1 + 2	57	2881.76	19.78	0.77 (0.59–1.01)	0.82 (0.63–1.07)	0.8 (0.61–1.05)
1 + 3	3	149.64	20.05	0.84 (0.27–2.61)	0.83 (0.27–2.57)	0.84 (0.27–2.61)
2 + 1	5599	255,090.73	21.95	0.91 (0.84–0.98)*	0.88 (0.82–0.95)*	0.91 (0.84–0.98)*
2 + 2	1233	74,638.36	16.52	0.67 (0.61–0.74)**	0.73 (0.66–0.8)**	0.77 (0.71–0.85)**
2 + 3	55	3783.38	14.54	0.57 (0.44–0.76)**	0.72 (0.55–0.94)*	0.77 (0.58–1.01)
3 + 1	10,470	373,887.36	28.00	1.12 (1.04–1.21)*	0.92 (0.86–1)*	0.96 (0.88–1.03)
3 + 2	10,276	514,724.5	19.96	0.79 (0.73–0.86)**	0.79 (0.73–0.85)**	0.85 (0.79–0.92)**
3 + 3	964	59,349.6	16.24	0.64 (0.58–0.71)**	0.73 (0.66–0.81)**	0.79 (0.72–0.87)**
4 + 1	1515	39,514.3	38.34	1.45 (1.33–1.59)**	0.93 (0.85–1.02)	0.99 (0.9–1.08)
4 + 2	7589	290,091.26	26.16	1.02 (0.95–1.11)	0.89 (0.82–0.96)*	0.97 (0.9–1.05)
4 + 3	2173	107,941.76	20.13	0.8 (0.74–0.87)**	0.86 (0.79–0.94)**	0.96 (0.88–1.05)
5 + 1	140	3628.75	38.58	1.47 (1.23–1.77)**	0.78 (0.65–0.94)*	0.71 (0.59–0.85)**
5 + 2	2928	88,804.62	32.97	1.26 (1.16–1.37)**	0.95 (0.88–1.04)	1.04 (0.96–1.13)
5 + 3	2770	106,548.37	26.00	1.02 (0.94–1.11)	1.01 (0.93–1.09)	1.1 (1.01–1.2)*
6 + 1	12	168.21	71.34	2.55 (1.44–4.52)*	1.16 (0.65–2.05)	1.37 (0.77–2.43)

Note: Abbreviations: SBP = blood pressure; HR = hazard ratio; CI = confidence interval. Grade 1 ≤ 100 mmHg; Grade 2 = 100–119 mmHg; Grade 3 = 120–139 mmHg; Grade 4 = 140–159 mmHg; Grade 5 = 160–179 mmHg; Grade 6 ≥ 180 mmHg. Class 1 = Blood pressure gradually increases from ideal values with age; Class 2 = Blood pressure gradually increases from normal-high values with age; Class 3 = Blood pressure gradually increases from mild hypertension with age. ^a^Model 1 was a univariate analysis. ^b^Model 2 was adjusted for sex and age. ^c^Model 3 included variables in Model 1 and was further adjusted for marital status, educational level, tobacco smoking, alcohol drinking, physical activity, 7-year average waist circumference, 7-year average fasting blood glucose, 7-year average total cholesterol, and 7-year average body mass index. *P < 0.05; **P < 0.01.

### 3.5. Sensitivity analyses

Sensitivity analyses, including subgroup analyses by antihypertensive medication use (Figure S3), confirmed the robustness of the findings across different classifications of SBP (categorical/continuous) and mortality outcomes (all-cause, cardiovascular, cancer, other diseases).

## 4. Discussion

Our analysis of a natural population cohort of over 390,000 elderly people aged 65 and older in this study showed a nonlinear correlation between baseline SBP, 7-year mean SBP, and all-cause mortality. The correlation analysis between baseline SBP, 7-year mean SBP, and all-cause mortality rate in the study showed that the risk of mortality was relatively high when SBP was less than 100 mmHg and higher than 160 mmHg. When the baseline SBP was between 100 and 139 mmHg and increased with age, the risk of all-cause mortality in the elderly was lower.

The target blood pressure value for elderly people has always been controversial [1],[17],[18]. Some studies supported a strict control of blood pressure in the elderly, while others suggested that overly strict blood pressure control may be harmful to the health of the elderly [19]. The most reported cases were among frail elderly individuals who may require higher SBP, which may have a protective effect on all-cause mortality [19],[20]. A study in a primary care population aged above 75 showed that blood pressure <130/80 was associated with higher mortality [19]. There were also studies showing that elderly individuals with relatively high SBP and reduced functional and cognitive status also have lower mortality rates [21], but some studies have shown different perspectives [6],[22]. Therefore, some researchers stated that the elderly need to decide how to control blood pressure based on individual circumstances [23]. For the literature that provided recommendations on blood pressure values for the elderly, the conclusions vary, with corresponding recommendations for SBP < 120 mmHg, 130–139 mmHg, or 140–159 mmHg [24],[25]. Our article analyzed the relationship between baseline SBP, 7-year mean SBP, 7-year SBP trajectory, and overall cause of death, and suggested that the range of SBP values in elderly people should be above 100 mmHg to below 160 mmHg.

Single or short-term observations may lead to inaccurate data and fail to reflect long-term changes in individuals. Regarding the ability of the SBP trajectory to predict mortality, there were reports that long-term changes in SBP were an independent predictor of mortality, and SBP has greater significance for elderly people compared to diastolic blood pressure [26]. At present, there have been some reports on the relationship between SBP trajectory and disease outcomes [27],[28]. Most reports indicated that a decrease in blood pressure trajectory in the elderly was associated with a higher risk of all-cause mortality and cardiovascular disease mortality compared to stable trajectories at high normal levels [27],[29], and stable SBP levels in the moderate range were associated with lower mortality rates in the elderly [30]. Some elderly people experienced an accelerated decline in their SBP trajectory in the last two years of life without controlling their blood pressure [26]. This study showed that, after adjusting for sex, age, mental status, educational level, tobacco smoking, alcohol drinking, physical activity, 7-year WC, 7-year TC, 7-year FBG, and 7-year BMI, there was a 7-year SBP trajectory classified as Class 2 (where blood pressure gradually increased from the normal-high value with age, and all-cause mortality was the lowest), Class 1 (where blood pressure gradually increased from the ideal value with age, and cardiovascular mortality was the lowest), and Class 3 (where blood pressure gradually increased from mild hypertension with age, with the lowest mortality rates from cancer and other diseases other than cardiovascular and cancer). The combined analysis of 7-year SBP trajectory and baseline SBP showed that older adults with a baseline SBP between 100 and 139 mmHg (Grades 2 and 3) with an ideal blood pressure or normal-high value (Grade 2 and Grade 3) at 65 years of age and a progressively increasing SBP trajectory with age (Class 2 and Class 3) were at lower risk in older adults.

One of the reasons why the target blood pressure of elderly people needs to be comprehensively considered is the fact that the tolerance of various organs or functions throughout the body to blood pressure may vary. Some studies suggested that the heart may require higher blood pressure. However, in clinical practice, the relationship between cardiac surgery and blood pressure targets was also unclear [31],[32]. The relationship between cognitive function and blood pressure levels in elderly people has also been reported differently [33],[34]. This study also showed that all-cause mortality, cardiovascular mortality, cancer mortality, and mortality from diseases other than cardiovascular and cancer were also different for different baseline SBP combined with 7-year SBP trajectories.

This study has several strengths. We conducted a large-scale cohort study in a natural population aged 65 years and above, followed them for 7 years, and recorded their SBP data annually, which allowed us to accurately observe the trajectory of SBP and study the relationship between the dynamic changes of SBP and the risk of death, which provides an important reference for the prevention and control of hypertension, especially in the relatively economically underdeveloped areas of western China. In addition, this study conducted annual physical examinations for the elderly in the entire city of Luzhou, China, aiming to avoid selection bias caused by sampling surveys.

Several limitations should be considered when interpreting our findings. First, there were many reports indicating that there may be other better indicators reflecting changes in blood pressure, such as dynamic blood pressure, nighttime blood pressure, mean arterial pressure, moderate blood pressure, etc., which may better explain the risk of all-cause and cardiovascular death than clinical blood pressure [35]–[38]. Due to limitations in conditions, the measurement of blood pressure changes mentioned above has not been included in the measurement of this study. Second, although we divided the 7-year SBP trajectory into three categories, we did not differentiate the magnitude of SBP changes in more detail, which may affect our results and interpretation. Next, we will consider further exploring more detailed grouping and incorporating more mortality outcome variables. Third, most of the covariates we included were measured at baseline and cannot reflect the impact of changes during follow-up. Fourth, many elderly people were in a comorbid state, and their disease status has not yet been classified. Fifth, this study was based on a Chinese population, and there may be ethnic or regional differences. Future research needs to further classify and clarify the physical condition of the elderly, as well as conduct further validation in other ethnic groups or regions, in order to provide more in-depth guidance for the formulation of SBP target values for the elderly.

## 5. Conclusions

In summary, the 7-year prospective cohort results suggested that older adults with SBP rising with age from normal to stage 1 hypertension (no more than 160 mmHg), or even higher, from age 65, may be associated with lower mortality.

## Use of AI tools declaration

The authors declare they have not used Artificial Intelligence (AI) tools in the creation of this article.


