# Supplementary material for: Baseline and 7-Year longitudinal trajectories of systolic blood pressure and all-cause mortality in the elderly: A large prospective cohort study in China, 2017–2023
Source: AIMS Public Health. 2025 Aug 29;12(3):857–74. doi: 10.3934/publichealth.2025043 (PMC12538236; doi:10.3934/publichealth.2025043)
Supplement: Supplementary file 1 [file publichealth-12-03-043-s001.pdf]

Public Health

AIMS Public Health, 12(3): 857–874.
DOI: 10.3934/publichealth.2025043
Received: 23 June 2025
Revised: 18 August 2025
Accepted: 20 August 2025
Published: 29 August 2025

http://www.aimspress.com/journal/aimsph

Research article

Baseline and 7-Year longitudinal trajectories of systolic blood pressure and all-cause mortality in the elderly: A large prospective cohort study in China, 2017–2023

Zhuo Wang[1,†], Yujin He[1,†], Tianyong Wu[2,†], Zifang Zhou[3], Zhi Lei[2], Rujun Liao[1], Fangyi Yu[4], Pengyuan Yang[4], Xiaoyan Wang[2], Ling Liu[2], Longling Yang[2], Qing Yang[2], Xuefeng Tang[1], Xianping Wu[1], Bo Zhong[1,*], Hang Chen[2,*] and Peng Yin[3]

[1] Department of Chronic and Non-communicable Disease Control and Prevention, Sichuan Center for Disease Control and Prevention, Chengdu, China
[2] Department of Chronic and Non-communicable Disease Control and Prevention, Luzhou Center for Disease Control and Prevention, Luzhou, China
[3] National Center for Chronic and Non-communicable Disease Control and Prevention, Chinese Center for Disease Control and Prevention, Beijing, China
[4] School of Public Health, North Sichuan Medical College, Nanchong, China

* **Correspondence:** Bo Zhong: Email: zhongbo1969@163.com; Tel: +862885587325; Hang Chen: Email: 331999258@qq.com; Tel: +8608302584041.

† These three authors contributed equally.

Supplementary

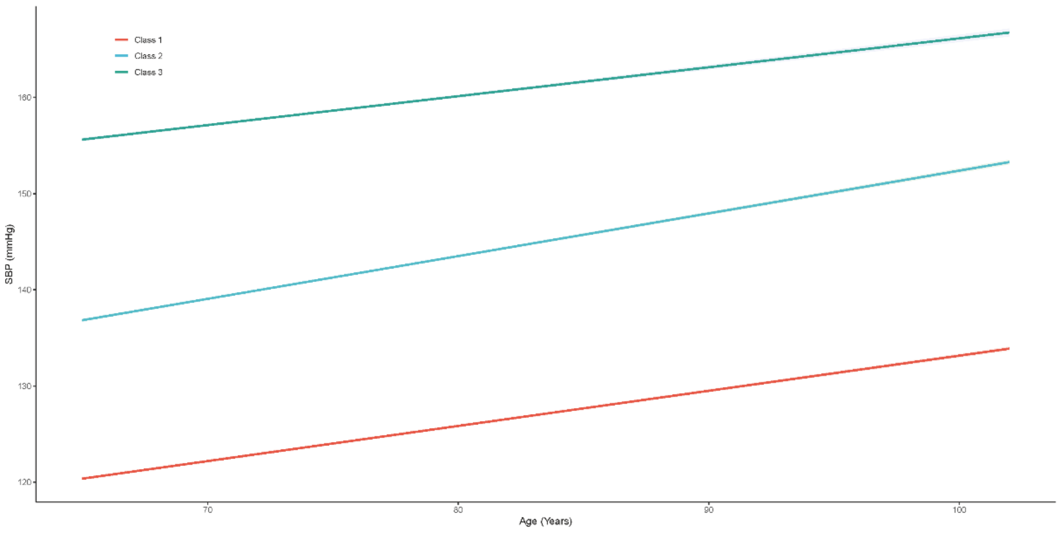

Figure S1. The trajectory of systolic blood pressure in the elderly for 7 years, Luzhou, China, 2017–2023. Note: Class 1 = Blood pressure gradually increases from ideal values with age; Class 2 = Blood pressure gradually increases from normal-high values with age; Class 3 = Blood pressure gradually increases from mild hypertension with age. Abbreviations: SBP = systolic blood pressure.

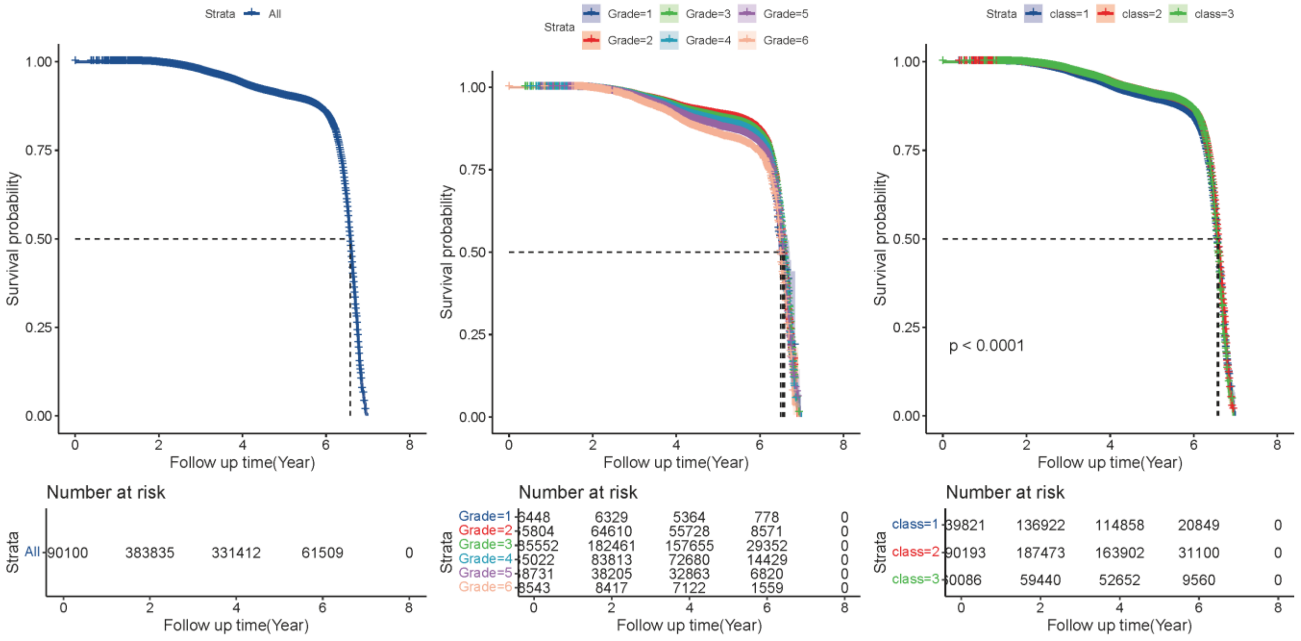

Figure S2. Kaplan-Meier overall survival estimates, comparison of mortality in participants stratified by SBP trajectories and. Note: Grade 1 ≤ 100 mmHg; Grade 2 = 100–119 mmHg; Grade 3 = 120–139 mmHg; Grade 4 = 140–159 mmHg; Grade 5 = 160–179 mmHg; Grade 6 ≥ 180 mmHg. Class 1 = Blood pressure gradually increases from ideal values with age; Class 2 = Blood pressure gradually increases from normal-high values with age; Class 3 = Blood pressure gradually increases from mild hypertension with age. Abbreviations: SBP = systolic blood pressure.

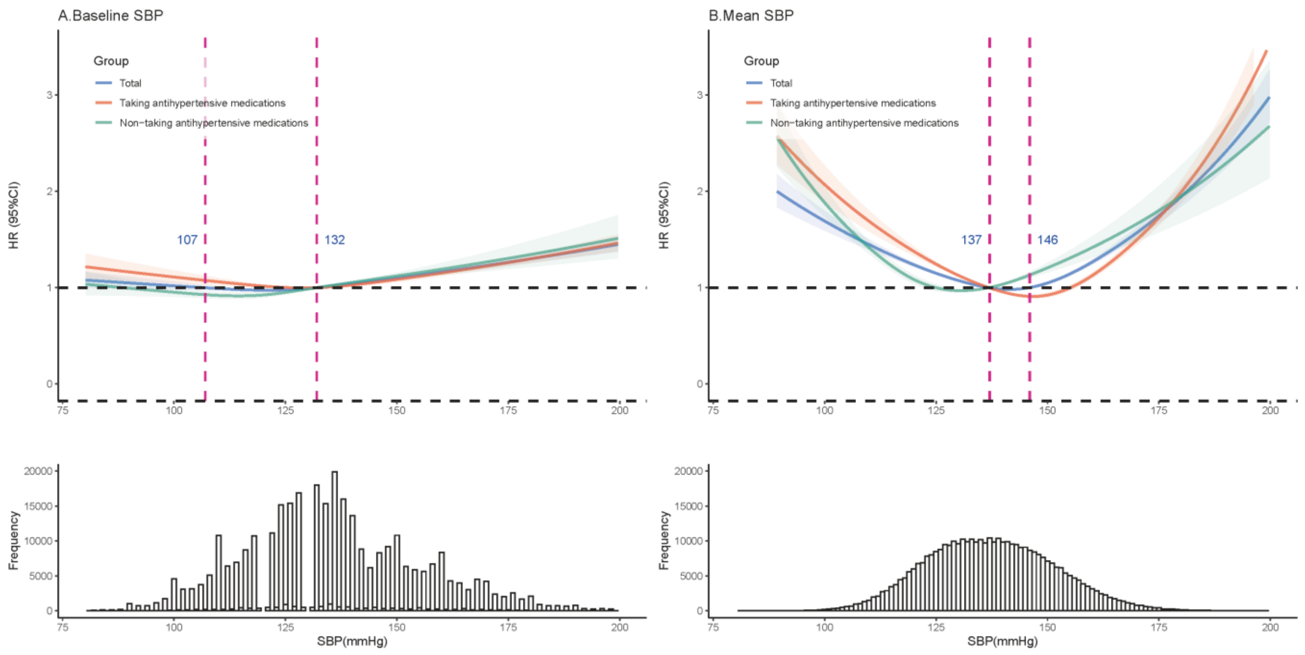

Figure S3. Association of adjusted baseline SBP and 7-year mean SBP as a continuous scale with mortality. Note: HRs (solid line) and 95% CIs (shadow) from Cox proportional hazards regression models using restricted cubic splines. Covariates included marital status, educational level, tobacco smoking, alcohol drinking, physical activity, 7-year average waist circumference, 7-year average fasting blood glucose, 7-year average total cholesterol, 7-year average body mass index. Graphs were truncated at 80 and 200 mmHg for more than 96% of participants were located between these cut points. Abbreviations: SBP = systolic blood pressure; CI = confidence interval.

Table S1. Definitions and measurement methods of questionnaire survey and physical measurement indicators.

Index	Frequency	Definition/measurement methods
Tobacco smoking	current	smoking at least one cigarette per day for continuously more than one year
	former	having previously smoked at least one cigarette per day for continuously more than one year, but having refrained from smoking continuously for more than one year as of the survey time
	never	As of the survey date, the respondent has never smoked any cigarette products
Alcohol drinking	everyday	drinking no fewer than 7 times a week, with at least one drink per day and each drink being no less than 100ml
	often	drinking liquor no fewer than 3 times a week, with each drink being no less than 100ml
	occasionally	drinking occurs less than 3 times per week
	never	having not consumed any alcohol-containing beverages or foods in the past year
Physical activity	less than once/week	engaging in moderate-intensity exercise lasting 30-60 minutes or vigorous-intensity exercise lasting 20-25 minutes with a frequency of less than once per week
	more than once/week	engaging in moderate-intensity exercise lasting 30-60 minutes or vigorous-intensity exercise lasting 20-25 minutes more than once but fewer than 7 times per week
	everyday	engaging in moderate-intensity exercise lasting 30-60 minutes or vigorous-intensity exercise lasting 20-25 minutes at least once per day
Blood pressure		Blood pressure measurements are uniformly performed using calibrated upper-arm electronic sphygmomanometers. Before measurement, the subject rests quietly in a sitting position for at least 5 minutes. Measurements are taken consecutively twice in a quiet state, with an interval of 1 minute, and the average of the two readings is recorded.
Height		Medical institutions are uniformly equipped with electronic height and weight scales. The person being measured stands on the scale, leans back against the measuring ruler, stands straight, and maintains a straight line through the head, buttocks, and feet. Measurements are taken consecutively twice, ensuring that the difference between the two measurements is within 0.5 cm.
Weight		Medical institutions are uniformly equipped with electronic height and weight scales. The person, after fasting, removes their shoes, wears light clothing, and stands correctly on the base of the electronic height and weight scale, with the body kept straight. The value displayed and recorded by the electronic scale is the person's weight.

Continued on next page

Index	Frequency	Definition/measurement methods
Waist circumference		Medical institutions are uniformly equipped with waist circumference measuring tapes. During the measurement, the subject stands with feet separated by 25 to 30 cm. The measurement position is the midpoint of the horizontal line connecting the anterior superior iliac spine and the inferior margin of the 12th rib. The measuring tape is placed snugly against the soft tissue without compressing it, and the measurement is accurate to 0.1 cm.
Total cholesterol		Venous blood is collected from subjects after 8–12 hours of fasting. Excessive greasy food should be avoided the day before the examination. During transportation, blood samples should be protected from severe shaking to prevent hemolysis. For distant collection sites, samples should be transported using 2–8℃ transport boxes. All medical institutions perform serum cholesterol, triglyceride, low-density lipoprotein, and high-density lipoprotein tests in strict accordance with the relevant procedures of the biochemical analyzers and supporting reagents used.
Fasting blood glucose		Venous blood is collected after 8–12 hours of fasting. During transportation, blood samples should be protected from severe shaking to prevent hemolysis. For distant collection sites, samples should be transported using 2–8℃ transport boxes. All medical institutions measure fasting blood glucose in strict accordance with the relevant procedures of the biochemical analyzers and supporting reagents used.

Table S2. Association of baseline SBP and 7-year SBP trajectories with cardiovascular mortality.

Group	Number of deaths	Follow-up duration (person-years)	Mortality rate (per 10,000 person-years)	Adjusted HR (95% CI)			
				Model 1[a]	Model 2[b]	Model 3[c]	Model 4[d]
Baseline SBP (mmHg)							
Grade1	234	32,431.31	7.22	NA	NA	NA	NA
Grade2	2198	333,512.47	6.59	0.91 (0.79–1.04)	0.9 (0.79–1.03)	0.91 (0.79–1.04)	0.89 (0.78–1.02)
Grade3	7909	947,961.46	8.34	1.11 (0.97–1.26)	1.02 (0.9–1.16)	1.03 (0.9–1.17)	0.96 (0.84–1.09)
Grade4	5152	437,547.32	11.77	1.54 (1.35–1.76)**	1.34 (1.18–1.53)**	1.37 (1.2–1.57)**	1.19 (1.04–1.36)*
Grade5	2970	198,981.74	14.93	1.94 (1.7–2.21)**	1.63 (1.43–1.86)**	1.66 (1.45–1.9)**	1.34 (1.17–1.55)**
Grade6	839	43,616.12	19.24	2.51 (2.17–2.9)**	2.07 (1.79–2.39)**	2.09 (1.81–2.42)**	1.6 (1.37–1.86)**
7-year SBP trajectories							
Class 1	5808	701,689.26	8.28	NA	NA	NA	NA
Class 2	9673	980,183.86	9.87	1.17 (1.13–1.2)**	1.28 (1.24–1.32)**	1.29 (1.25–1.34)**	1.15 (1.11–1.19)**
Class 3	3821	312,177.3	12.24	1.45 (1.39–1.51)**	1.8 (1.73–1.88)**	1.84 (1.76–1.91)**	1.4 (1.33–1.48)**

Note: Abbreviations: SBP = systolic blood pressure; HR = hazard ratio; CI = confidence interval. Grade 1 ≤ 100 mmHg; Grade 2 = 100–119 mmHg; Grade 3 = 120–139 mmHg; Grade 4 = 140–159 mmHg; Grade 5 = 160–179 mmHg; Grade 6 ≥ 180 mmHg. Class 1 = Blood pressure gradually increases from ideal values with age; Class 2 = Blood pressure gradually increases from normal high values with age; Class 3 = Blood pressure gradually increases from mild hypertension with age. [a]Model 1 was univariate analysis. [b]Model 2 adjusted for sex, age. [c]Model 3 included variables in model 1 and further adjusted for marital status, educational level, tobacco smoking, alcohol drinking, physical activity, 7-year average waist circumference, 7-year average fasting blood glucose, 7-year average total cholesterol, 7-year average body mass index. [d]Model 4 included variables in model 2 and further adjusted for SBP trajectories or SBP at baseline. *$P < 0.05$; **$P < 0.01$.

Table S3. Association of baseline SBP and 7-year SBP trajectories with cancer mortality.

Group	Number of deaths	Follow-up duration (person-years)	Mortality rate (per 10,000 person-years)	Adjusted HR (95% CI)			
				Model 1[a]	Model 2[b]	Model 3[c]	Model 4[d]
Baseline SBP (mmHg)							
Grade 1	156	32,431.31	4.81	NA	NA	NA	NA
Grade 2	1579	333,512.47	4.73	0.98 (0.83–1.15)	0.96 (0.82–1.13)	1 (0.85–1.18)	1.02 (0.86–1.2)
Grade 3	4945	947,961.46	5.22	1.04 (0.88–1.21)	0.98 (0.84–1.15)	1.05 (0.89–1.23)	1.15 (0.98–1.35)
Grade 4	2202	437,547.32	5.03	0.98 (0.84–1.16)	0.91 (0.77–1.07)	1 (0.85–1.18)	1.19 (1.01–1.41)*
Grade 5	1000	198,981.74	5.03	0.97 (0.82–1.15)	0.89 (0.75–1.05)	0.98 (0.82–1.16)	1.23 (1.03–1.47)*
Grade 6	213	43,616.12	4.88	0.95 (0.77–1.17)	0.86 (0.7–1.06)	0.95 (0.77–1.17)	1.25 (1.01–1.55)*
7-year SBP trajectories							
Class 1	4129	701,689.26	5.88	NA	NA	NA	NA
Class 2	4694	980,183.86	4.79	0.8 (0.76–0.83)**	0.82 (0.79–0.86)**	0.86 (0.82–0.9)**	0.82 (0.78–0.86)**
Class 3	1272	312,177.3	4.07	0.68 (0.64–0.72)**	0.74 (0.7–0.79)**	0.79 (0.74–0.84)**	0.72 (0.67–0.78)**

Note: Abbreviations: SBP = systolic blood pressure; HR = hazard ratio; CI = confidence interval. Grade 1 ≤ 100 mmHg; Grade 2 = 100–119 mmHg; Grade 3 = 120–139 mmHg; Grade 4 = 140–159 mmHg; Grade 5 = 160–179 mmHg; Grade 6 ≥ 180 mmHg. Class 1 = Blood pressure gradually increases from ideal values with age; Class 2 = Blood pressure gradually increases from normal high values with age; Class 3 = Blood pressure gradually increases from mild hypertension with age. [a]Model 1 was univariate analysis. [b]Model 2 adjusted for sex, age. [c]Model 3 included variables in model 1 and further adjusted for marital status, educational level, tobacco smoking, alcohol dr inking, physical activity, 7-year average waist circumference, 7-year average fasting blood glucose, 7-year average total cholesterol, 7-year average body mass index. [d]Model 4 included variables in model 2 and further adjusted for SBP trajectories or SBP at baseline. *P < 0.05; **P < 0.01.

Table S4. Association of baseline SBP and 7-year SBP trajectories with mortality from other diseases excluding cardiovascular and cancer.

Group	Number of deaths	Follow-up duration (person-years)	Mortality rate (per 10,000 person-years)	Adjusted HR (95% CI)			
				Model 1[a]	Model 2[b]	Model 3[c]	Model 4[d]
Baseline SBP (mmHg)							
Grade1	372	32,431.31	11.47	NA	NA	NA	NA
Grade2	3110	333,512.47	9.32	0.81 (0.72–0.9)**	0.8 (0.72–0.89)**	0.86 (0.77–0.96)*	0.88 (0.79–0.98)*
Grade3	8856	947,961.46	9.34	0.78 (0.7–0.86)**	0.71 (0.64–0.79)**	0.8 (0.72–0.89)**	0.89 (0.8–0.99)*
Grade4	3923	437,547.32	8.97	0.73 (0.66–0.81)**	0.63 (0.57–0.7)**	0.75 (0.68–0.84)**	0.93 (0.83–1.04)
Grade5	1868	198,981.74	9.39	0.76 (0.68–0.85)**	0.63 (0.56–0.71)**	0.75 (0.67–0.84)**	0.98 (0.87–1.1)
Grade6	487	43,616.12	11.17	0.91 (0.79–1.04)	0.74 (0.65–0.85)**	0.89 (0.78–1.02)	1.21 (1.05–1.4)*
7-year SBP trajectories							
Class 1	8501	701,689.26	12.12	NA	NA	NA	NA
Class 2	8049	980,183.86	8.21	0.66 (0.64–0.68)**	0.73 (0.7–0.75)**	0.79 (0.77–0.82)**	0.77 (0.75–0.8)**
Class 3	2066	312,177.3	6.62	0.53 (0.51–0.56)**	0.66 (0.63–0.7)**	0.75 (0.71–0.79)**	0.69 (0.65–0.73)**

Note: Abbreviations: SBP = systolic blood pressure; HR = hazard ratio; CI = confidence interval. Grade 1 ≤ 100 mmHg; Grade 2 = 100–119 mmHg; Grade 3 = 120–139 mmHg; Grade 4 = 140–159 mmHg; Grade 5 = 160–179 mmHg; Grade 6 ≥ 180 mmHg. Class 1 = Blood pressure gradually increases from ideal values with age; Class 2 = Blood pressure gradually increases from normal high values with age; Class 3 = Blood pressure gradually increases from mild hypertension with age. [a]Model 1 was univariate analysis. [b]Model 2 adjusted for sex, age. [c]Model 3 included variables in model 1 and further adjusted for marital status, educational level, tobacco smoking, alcohol dr inking, physical activity, 7-year average waist circumference, 7-year average fasting blood glucose, 7-year average total cholesterol, 7-year average body mass index. [d]Model 4 included variables in model 2 and further adjusted for SBP trajectories or SBP at baseline. *P < 0.05; **P < 0.01.

AIMS Press

© 2025 the Author(s), licensee AIMS Press. This is an open access article distributed under the terms of the Creative Commons Attribution License (http://creativecommons.org/licenses/by/4.0)
